# A Pragmatic Approach to Getting Published: 35 Tips for Early Career Researchers

**DOI:** 10.3389/fpls.2016.00610

**Published:** 2016-05-09

**Authors:** Natasha M. Glover, Ioanna Antoniadi, Gavin M. George, Lars Götzenberger, Ruben Gutzat, Kadri Koorem, Pierre Liancourt, Kinga Rutowicz, Krishna Saharan, Wanhui You, Philipp Mayer

**Affiliations:** ^1^Bayer CropScience Innovation CenterGent, Belgium; ^2^Department of Genetics, Evolution and Environment, University College LondonLondon, UK; ^3^Department of Forest Genetics and Plant Physiology, Umeå Plant Science Centre, Swedish University of Agricultural SciencesUmeå, Sweden; ^4^Institute of Agricultural Sciences, ETH ZurichZurich, Switzerland; ^5^Institute of Botany, Czech Academy of SciencesTřeboň, Czech Republic; ^6^Gregor Mendel InstituteVienna, Austria; ^7^Department of Terrestrial Ecology, Netherlands Institute of EcologyWageningen, Netherlands; ^8^Institute of Plant Biology, University of ZurichZurich, Switzerland; ^9^Department of Environmental Sciences - Botany, University of BaselBasel, Switzerland; ^10^Science-TextflowWinterthur, Switzerland

**Keywords:** publishing, early career researcher, collaboration, journals, open access, strategy

## Abstract

It is trite to say “publish or perish,” yet many early career researchers are often at a loss on *how* to best get their work published. With strong competition and many manuscripts submitted, it is difficult to convince editors and reviewers to opt for acceptance. A pragmatic approach to publishing may increase one's odds of success. Here, we – a group of postdocs in the field of plant science – present specific recommendations for early career scientists on advanced levels. We cannot provide a recipe-like set of instructions with success guaranteed, but we come from a broad background in plant science, with experience publishing in a number of journals of varying topics and impact factors. We provide tips, tricks, and tools for collaboration, journal selection, and achieving acceptance.

## Introduction

Publishing is essential for scientists to disseminate their research and communicate insights to as broad of an audience as possible. For early career scientists, in particular, publication record is the fundamental metric for judging their career advancement (Walker et al., 2010). However, with the overwhelming number of papers submitted to peer-reviewed journals, it is challenging to get your work noticed and stand out from the crowd. A strategic approach to publishing can maximize your chances of success.

Scientifically-robust research is a critical first step in getting any paper published. But what to do after that? General advice on getting published is available in various textbooks (Rosei and Johnston, 2006; Fraser, 2008; Albert, 2009; Davis et al., 2012; Cargill and O'Connor, 2013), articles (Bourne, 2005; Johnson and Green, 2009; Powell, 2010) and websites (Kelner, 2007; Gould, 2014). However, there is a shortage of brief, synoptic overviews and specific recommendations for early career scientists on advanced levels (e.g., postdocs).

Here, we present our pragmatic tips for advanced publication strategies. The recommendations were developed on September 28, 2015 at a workshop for PLANT FELLOWS, an international postdoc fellowship program in the field of plant sciences (www.plantfellows.ch). These suggestions are based on the experiences of the ten workshop participants, the instructor, and the literature.

We start with recommendations for collaboration, as research has become more and more of a joint effort. The number of partners and collaboration strategy has been shown to correlate strongly with a researcher's productivity, i.e., the number of published peer-reviewed journal articles (Lee, 2005). In the life sciences, collaborative papers result in more highly cited publications (Figg et al., 2006), and international collaborations appear to be especially fruitful (Adams, 2013).

We then provide advice for journal selection because submitting to an inappropriate journal will result in wasted time and effort. Unsuitable target journals could mean unnecessarily giving away impact points (Rosei and Johnston, 2006). In addition, the publishing landscape has changed in recent years and has become even more complex, with new publishing concepts (e.g., open access, electronic preprints) and an ever-increasing number of submitted and published articles (Changing Nature, 2013). This dynamic publishing landscape makes it important to target the right journals for your research.

Since acceptance is the ultimate aim of any submitted manuscript, lastly we give tips for achieving acceptance of your paper.

## Collaboration

Collaboration is important for many reasons. From a scientific standpoint, collaborations enable us to find innovative solutions to interdisciplinary problems, such as those related to climate change, public health, or the energy sector (Adams, 2012). A good collaboration can provide fresh input, intellectual stimulation, and motivation. From a practical standpoint, collaborating with people from other fields can facilitate your movement to other disciplines, and enables access to ideas, resources, and partners' facilities (Gabrys and Langdale, 2012). In the plant sciences, access to greenhouses, fields, labs, and high-performance computing clusters may be shared. Collaborations are a way to network, establish yourself, and demonstrate your skills and work ethic (Dee, 2006). From a strategic point of view, collaboration allows for partners to publish in a synergetic way (back to back papers/companion papers) and thus avoid competition (Dee, 2006; Gabrys and Langdale, 2012).

### There are multiple ways to establish collaborations

1. Make contact with potential collaborators at conferences. Participation in coffee breaks and dinners are just as important as the scientific sessions. However, the “big shots” are often surrounded by people and may not remember you. Thus:
2. Contact potential partners via email. This may be preferable for some potential collaborators as it does not demand immediate reaction. Use an eye-catching subject line and well thought-out message to make sure your email gets noticed (Dee, 2006).
3. Put yourself out there. Do publicity for your previous publications to make you and your research visible to the scientific community (npg, 2015). Presenting your work at a conference may inspire potential collaborators to contact you. There are special grants for setting up initial meetings/workshops for collaborators, for example European Cooperation in Science and Technology (www.cost.eu). Look into your local grant opportunities that promote collaborative efforts.
4. Collaborations between industry (biotechnology companies for example) and academia can come with numerous benefits for both parties including networking, recruitment possibilities, financial support, and access to “novel techniques and concepts” (Pronk et al., 2015). Although there will likely be some degree of restriction on publication while working with a company due to confidentiality issues, there are possibilities to publish research resulting from the collaboration. For example, review papers or research papers using public rather than private data are publishing options.

### Successful collaborations need nurture

1. Be open minded to accept partners' ideas and views (Adams, 2012). Treat all collaborators as equals and with respect.
2. Good communication between all parties at all stages is crucial. Today, there are many collaboration tools to facilitate communication– everything from video conferences, instant messaging, file sharing, online meeting notes, assigning tasks, etc. (Wikipedia, 2015). We give our favorites in Table 1.
3. However, keep in mind that international collaborations can lead to issues or misunderstandings due to language barriers, especially in electronic format. We find that engaging in face to face meetings—at least periodically—can be beneficial for overall communication. Video conferences or Skype sessions might also be helpful. If some confusion arises due to language, don't hesitate to ask for clarification.
4. Set the rules of the game in the beginning. Designate a project coordinator and decide who will do what. Meet before the research to agree on ethical standards, plan of action, outcomes, journals for publication, data ownership, and authorship. It may be wise to choose the first author at the beginning and to decide on an author evaluation approach to implement downstream (Tscharntke et al., 2007). Make sure there are clear expectations at the outset (Gabrys and Langdale, 2012); see Smalheiser et al. (2005) for guidelines for negotiating a collaboration, and the Montreal Statement on Research Integrity in Cross-Boundary Research Collaborations (2013) for guidelines for individual and institutional responsibilities. For industry-academic collaborations, make sure to have a clear and contractual agreement ahead of time for expected IP and publication outcomes.

**Table 1:** **Recommended software tools for collaborative research**.

**Function**	**Tool**	**Website**
Videoconference	BigBlueButton^*^ GoToMeeting Skype^*^ WebEx	www.bigbluebutton.org www.gotomeeting.com www.skype.com www.webex.com
Document sharing/editing	Authorea^*^ Google Docs^*^ Overleaf^*^	www.authorea.com www.google.com/docs www.overleaf.com
File sharing	Asana^*^ Atlassian Confluence Dropbox^*^ Google Drive^*^ Tresorit	www.asana.com www.atlassian.com www.dropbox.com www.google.com/drive www.tresorit.com
Bibliography sharing	Colwiz^*^ EndNote Online^*^ Mendeley^*^ Paperpile Zotero^*^	www.colwiz.com www.myendnoteweb.com www.mendeley.com www.paperpile.com www.zotero.org
Group calendars	Asana^*^ Atlassian Confluence Google Calendars^*^ Trello^*^	www.asana.com www.atlassian.com www.google.com/calendar www.trello.com

* *Free or freemium software*.

### Be careful

1. Commitment and enthusiasm are needed from everyone. Collaborations can all too often devolve into *groupthink* (when the desire of conformity in a group of people leads to irrational decision-making, Janis, 1972) or the *Ringelmann effect* (when individuals in a group put in less and less effort as the size of the group increases, Simms and Nichols, 2014). Be aware of interpersonal, societal, and political factors that could inhibit good collaborations (Stokols et al., 2008).
2. Do not spread yourself too thin, especially by having too many projects in which you are a middle-author. Try to use the collaboration to gain experience and next time become a leader.
3. Collaborating with “big names” can be good, as long as it is within ethical guidelines– They must significantly contribute to the project to justify authorship. See Clement (2014) for an authorship framework and Wager and Kleinert (2011) for the Committee on Publication Ethics (COPE) standards for authors. Try to learn from their experience and use their network. Finally, make sure that big names do not get all the credit: Publish some papers without them to show your independence as a researcher.

## Journal selection

In your PhD and postdoc years, time is a precious resource, and it is important to choose the most appropriate journal to submit to. Although impact factor arguably should not be the *be all and end all* (Verma, 2015), many research institutions/funding agencies consider publication in high impact factor journals when hiring or giving out grants (Casadevall and Fang, 2014). The “right” journal will increase your research's visibility and build your reputation, whereas submitting to the “wrong” journal could decrease your chances of obtaining an academic job.

### A good fit between your research and the journal is of key importance

1. Make sure that the scope of the potential journal matches your paper. Knowing how your research fits in with the mission of the journal can help you avoid formatting and editing a paper, only for it to be rejected by the editor because it is outside the scope of the journal. Many plant-specific journals have a broad scope, publishing papers from nearly all aspects of plant science. For example, *Plant Science, Nature Plants, Frontiers in Plant Science*, and *Plant Physiology* all publish papers on topics including (but not limited to) cell biology, development, genetics, genomics, and physiology. Others have a more targeted audience (e.g., *Plant Pathology, Weed Science, Tree Physiology*). We have listed the top 30 journals in plant science, ranked by their SCImago Journal Rank for 2014, and indicated which sub-disciplines are covered within their scope (Table 2).
2. One helpful technique for choosing the right journal: check in which journals your cited references have been published in. This way you can rapidly assess which journals have previously accepted articles about your subject matter. There are online tools available where you can input the title and abstract of your manuscript and scan it on the web, resulting in a list of the journals that would be a good fit for your research. Examples of such kind of websites are: https://www.journalguide.com, https://www.edanzediting.com/journal-selector, http://www.biomedcentral.com/submissions/find-the-right-journal, http://jane.biosemantics.org, and http://journalfinder.elsevier.com.
3. Be realistic with the quality of your research. Unfortunately, many traditional journals judge the “quality” of the research by the expected citation rate and impact (Aarssen et al., 2010). Everyone wants to publish in high impact factor journals, however due to the sheer number of submissions, the majority are rejected. For example, *Nature* received 10,952 manuscripts in 2013, but only published 7.8% of them (Getting published in Nature : the editorial process^2^). Hence try to select a journal corresponding to the impact level of your work; see Aarssen et al. (2010) for a guide to evaluating the quality of your research.

**Table 2:** **Top 30 Plant Science journals, as determined by their SCImago Journal Rank (http://scimagojr.com)**.

**Rank**	**Title**	**SJR**	**2014 Impact Factor**	**not plant-specific**	**Agriculture**	**Biochemistry**	**Bioinformatics**	**Cell Biology**	**Development**	**Ecology**	**Evolution**	**Genetics**	**Genomics**	**Metabolism**	**Molecular biology**	**Physiology**	**Plant-microbe interactions**	**Proteomics**	**Systems biology**
1	Annual Review of Pathology: Mechanisms of Disease	11.47	18.75	x													x		
2	Annual Review of Plant Biology	10.69	18.90			x			x			x	x		x				
3	Trends in Plant Science	5.72	13.48		x	x		x	x	x	x	x	x	x	x	x	x	X	x
4	Studies in Mycology	5.05	13.25	x															
5	Annual Review of Phytopathology	4.93	9.62		x					x							x		
6	Plant Cell	4.83	9.34			x		x	x		x	x			x				
7	Current Opinion in Plant Biology	4.39	7.85					x	x			x	x	x	x	x	x		
8	Plant Physiology	3.42	6.84			x		x				x			x	x			
9	BMC Biology	3.27	7.43	x															
10	Plant Journal	3.24	5.97			x		x				x			x	x			
11	Journal of Ecology	3.02	5.43							x							x		
12	New Phytologist	2.86	7.67			x		x	x	x	x	x	x		x	x	x		
13	Molecular Plant	2.79	6.34			x	x	x	x		x	x	x		x	x	x		
14	Persoonia	2.57	5.30	x															
15	Plant, Cell and Environment	2.34	6.96			x		x		x		x			x	x			
16	Journal of Experimental Botany	2.31	5.53					x	x	x		x	x	x			x		
17	Plant and Cell Physiology	2.19	4.93			x	x	x				x	x		x	x	x		
18	Fungal Diversity	2.16	6.22	x															
19	Plant Biotechnology Journal	2.03	5.75			x	x		x			x	x	x	x	x		X	
20	Plant Molecular Biology	1.76	4.26			x	x						x		x			X	
21	Critical Reviews in Plant Sciences	1.74	5.44		x	x		x		x		x			x	x			
22	Perspectives in Plant Ecology, Evolution and Systematics	1.67	3.61							x	x								
23	Molecular Plant Pathology	1.65	4.72												x		x		
24	Journal of Vegetation Science	1.63	3.71							x									
25	Integrative and Comparative Biology	1.62	2.93	x															
26	Frontiers in Plant Science	1.55	3.95		x		x	x	x	x	x	x	x	x	x	x	x	X	x
27	Plant Methods	1.54	3.10			x		x				x	x		x				
28	Fungal Biology Reviews	1.53	N/A	x															
29	BMC Plant Biology	1.51	3.81			x		x	x		x	x	x		x	x	x		
30	Plant Genome	1.48	3.93										x						

*SJR (Scimago Journal Rank) refers to the “average number of weighted citations received in the selected year by the documents published in the selected journal in the three previous years” (SJR - Help ^1^). The subdisciplines for each journal were taken from their website. The “not plant-specific” column means the journal was listed as a plant science journal in SJR, however its scope is broader. Database accessed March 7, 2016*.

### Find the balance between impact factor and rejection rate

1. In some disciplines, high impact factor journals have high rejection rates. For example, Aarssen et al. (2008) found that the impact factor of ecology journals was directly correlated with rejection rate. However, for lower impact factor ecology journals the relationship is less obvious: some low impact factor journals have high rejection rates. Another analysis, based on 570 randomly selected journals, found little to no correlation between impact factor and rejection rate (Rocha da Silva, 2015), even when looking at only life science journals (Rocha da Silva, 2016). Thus, be cautious that some journals provide relatively less “bang for your buck” in terms of impact vs. rejection rate (Aarssen et al., 2008).
2. Be aware that studies published in journals with too low an impact factor may not be considered “good” research and could lead to lower evaluations by job search committees or funding agencies. However, this varies from field to field.

### Consider time from submission to publication

1. Consider the decision time of target journals, especially if you are under time pressure (e.g., strong competition, obligations to funders, or approaching the end of your project). Refer to www.journalguide.com for decision and publication speeds.
2. Take information provided by journals about their decision and publication speed with a grain of salt. Many papers are “soft” rejected and require resubmission, making the decision time appear to be short. Consult your peers to get a more realistic estimate of start to finish (see also www.scirev.sc).
3. For individual and unpredictable reasons (e.g., difficulties to find reviewers), the review process can be unusually long. If the decision is taking longer than promised at the time of submission, do not hesitate to send a friendly inquiry email to the managing editor.

### Think about open access in addition to traditional publication routes

1. Open access (OA) journals have been on the rise in the past decade (Björk and Solomon, 2012). These open access journals are establishing themselves as high-quality research outlets (Björk and Solomon, 2012) and their reputations have significantly improved in recent years (Changing Nature, 2013; Bourke-Waite, 2015). If you've dismissed OA journals before as being low-quality, it may be time to reconsider them.
2. Several peer-reviewed OA publishers or megajournals such as *PLOS ONE, BioMed Central* (*BMC* series) and the *Frontiers in* series cover a broad range of topics and publish high quality research, including plant science. *Frontiers in Plant Science* or *BMC Plant Biology* are examples of plant-specific journals from these publishers. In addition to being OA, these journals focus on the scientific soundness of the article, rather than the potential impact. Thus, their acceptance rate is generally higher than that of traditional journals and it may be easier to get your paper accepted (Björk, 2015; Walker and Rocha da Silva, 2015).
3. There is not yet a clear indication if publishing in OA journals is beneficial for career advancement (Antelman, 2004; Craig et al., 2007; Haug, 2013). Nevertheless, OA journals potentially accrue more citations due to their wider availability (Antelman, 2004; Pontika, 2015), which will ultimately increase your H-index. For example, a study of OA articles in the agricultural sciences showed double the citation rate compared to non-OA papers (Kousha and Abdoli, 2010).
4. With the rise of OA journals in recent years, the line between OA and traditional journals has become blurred. Many traditional subscription-based journals offer OA options for individual articles (e.g., *Current Opinion in Plant Biology, The Plant Journal*, and *The Plant Cell*). Thus, it's possible to take advantage of OA benefits while at the same time publishing in a highly-regarded journal.
5. Be aware of OA publishing outlets that have no adequate peer review system for quality control. There are a few warning signs which can help recognize a predatory publisher. For example, unsolicited spam emails to request submissions, an extremely fast decision to publish, and not receiving the final peer reviewer comments are big red flags (see Butler, 2013, and Beal's list of potential predatory publishers: http://scholarlyoa.com/publishers/).
6. Preprints are scientific articles that are not yet peer-reviewed, but published on an online database such as ArXiv (http://arxiv.org), bioRxiv (http://biorxiv.org), and PeerJ Preprints (https://peerj.com) (Desjardins-Proulx et al., 2013). Preprints may be a good idea to get your work out to the scientific community as soon as possible. Furthermore, you can often first preprint, then publish in a traditional journal. Although some traditional journals do not accept already-published material, many others like *Current Plant Biology, The Plant Cell, Nature*, and *Science* allow for submission of prepublished papers. See Sherpa/Romeo (http://sherpa.ac.uk/romeo/index.php) for a searchable database of preprint policies by journal.

## Achieving acceptance

Generating quality research is, of course, crucial for the success of any project, but the concise presentation of that work is critical for its impact, longevity and citability. However, it is a common misconception among early career researchers that the presentation of the work in a manuscript is the last stage of a project. There is a long and complicated process associated with submission, review, and revision that must be taken into account.

### Initial contact with the editor is important

1. Make sure your article is well-written. Editors will usually read the manuscript before sending it out for peer review to make sure it is within the scope of the journal and has good language and structure (Cargill and O'Connor, 2013). Therefore, spelling and grammar mistakes must be kept to a minimum. Get a native English speaker to proofread your manuscript. Additionally, writing with clarity, good argumentation, and a logical flow of ideas are key factors for the editor to understand your paper and be persuaded to send it out for review (Powell, 2010; Davis et al., 2012; Benson and Silver, 2013).
2. As the first contact between you and the editor, a thought-out cover letter is crucial. Some journals, such as the *New Phytologist*, require certain questions to be answered in the cover letter. Always read the journal's website for specific requirements. As a bare minimum, include a brief description of what your paper is about and why it is important in the context of your field. See Hafner (2010) and Doerr (2013) for some Dos and Don'ts for writing a cover letter.
3. As an author, you will always have a bias toward the importance of your work (Johnson and Green, 2009). In general, it is good practice to lean toward modesty in scientific writing. On the other hand, it is critical to highlight the novelty and importance in the article and cover letter in order to be published. You must balance out these two competing forces and present a body of work which is factual and well-supported.
4. A good practice is to put yourself in the place of the editor or reviewer, re-read your work again and try to be as objective as possible. This is true not just for the manuscript, but for all communications with the editor and/or reviewers.

### Understand the process and fight for your work

1. Once you submit a paper, one editor (or a few editors) check it before sending it off for expert review. Between two to four experts review the paper and send back comments which are compiled by the editor(s) and used to make a decision on whether to publish, send back for revision, or reject. Be prepared for discrepancies between reviewers opinions and for diverging suggestions for manuscript improvement.
2. Generally, an effective strategy is to go point by point through the reviewer comments and either make the suggested changes or politely explain and clarify the misunderstandings. If there is a legitimate disagreement between you and the reviewer, back up your point of view with other research from the literature. Typical ways to address the reviewer concerns include:
  - “You are right, here's how we addressed the criticism…”
  - “This is a misunderstanding because things were unclearly formulated. We have clarified…”
  - “This is a legitimate/pertinent concern in general, but in this specific case…”
3. It is important to realize that the decision lies ultimately with the editor. If you feel like the reviewers (or the editor) were unjust or misunderstood your work, make your case to the editor.

### Remove your personal feelings from the peer review process

1. When you have spent years creating something it is extremely difficult not to take peer criticism personally (Annesley, 2011). It is important to keep in mind that the function of peer review is not only to correct mistakes but to improve the quality of your work. Reviewers help authors.
2. Suggestions and questions by reviewers represent those that may be asked by your general audience and, once addressed, should increase the value of your work. Systematically address each of the reviewer concerns in a polite manner.
3. Do not forget the random component. Due to unforeseen/subjective factors, you still might need a bit of luck to get accepted.

## Conclusions

The foundation for getting your work published is by first conducting high-quality research. But even after this, getting published is a complex issue. The publishing landscape is constantly changing, so be willing to adapt your strategy. Even though many factors are out of the control of authors, various approaches are possible and it makes sense to make conscious and well-informed moves. Collaborating with researchers within as well as outside your field, sending your research article to an appropriate journal, and adequately highlighting the novelty and impact of your research can help pave the way to scientific recognition. The demanding task of implementing these recommendations in your own publishing strategy is left to you. Good luck.

## Author contributions

NG, IA, GG, LG, RG, KK, PL, KR, KS, WY, and PM all contributed to the brainstorming, writing, and critical review of this manuscript. NG and PM edited the manuscript.

### Conflict of interest statement

The authors declare that the research was conducted in the absence of any commercial or financial relationships that could be construed as a potential conflict of interest.

## Footnotes

^1^^1^SJR - Help Available at: http://scimagojr.com/help.php [Accessed March 14, 2016].

^2^Getting published in Nature : the editorial process Nat. Authors Referees. Available at: http://www.nature.com/nature/authors/get_published/ [Accessed October 22, 2015].

## References

[B1] Aarssen L. W. Lortie C. J. Budden A. E. (2010). Judging the quality of our research: a self-assessment test. Web Ecol. 10, 23–26. 10.5194/we-10-23-2010

[B2] Aarssen L. W. Tregenza T. Budden A. E. Lortie C. J. Koricheva J. Leimu R. (2008). Bang for your buck: rejection rates and impact factors in ecological journals. Open Ecol. J. 1, 14–19. 10.2174/1874213000801010014

[B3] Adams J. (2012). Collaborations: the rise of research networks. Nature 490, 335–336. 10.1038/490335a 23075965

[B4] Adams J. (2013). Collaborations: the fourth age of research. Nature 497, 557–560. 10.1038/497557a 23719446

[B5] Albert T. (2009). Winning the Publications Game: How to Write A Scientific Paper Without Neglecting Your Patients. Abingdon: Radcliffe.

[B6] Annesley T. M. (2011). Top 10 tips for responding to reviewer and editor comments. Clin. Chem. 57, 551–554. 10.1373/clinchem.2011.162388 21282666

[B7] Antelman K. (2004). Do open-access articles have a greater research impact? Coll. Res. Libr. 65, 372–382. 10.5860/crl.65.5.372

[B8] Benson P. J. Silver S. C. (2013). What Editors Want: An Author's Guide to Scientific Journal Publishing. Chicago : IL; London: University of Chicago Press.

[B9] Björk B.-C. (2015). Have the “mega-journals” reached the limits to growth? PeerJ 3:e981. 10.7717/peerj.981 26038735 PMC4451030

[B10] Björk B.-C. Solomon D. (2012). Open access versus subscription journals: a comparison of scientific impact. BMC Med. 10:73. 10.1186/1741-7015-10-73 22805105 PMC3398850

[B11] Bourke-Waite A. (2015). Perceptions of Open Access Publishing are Changing for the Better, a Survey by Nature Publishing Group and Palgrave Macmillan Finds. Available online at: http://www.nature.com/press_releases/perceptions-open-access.html [Accessed October 14, 2015].

[B12] Bourne P. E. (2005). Ten simple rules for getting published. PLoS Comput. Biol. 1:e57. 10.1371/journal.pcbi.0010057 16261197 PMC1274296

[B13] Butler D. (2013). Investigating journals: the dark side of publishing. Nature 495, 433–435. 10.1038/495433a 23538810

[B14] Cargill M. O'Connor P. (2013). Writing Scientific Research Articles: Strategy and Steps, 2nd Edn. Chichester; West Sussex; Hoboken, NJ: Wiley-Blackwell John Wiley & Sons, Ltd., Publication.

[B15] Casadevall A. Fang F. C. (2014). Causes for the persistence of impact factor mania. mBio 5, e00064–14. 10.1128/mBio.01342-14 24643863 PMC3967521

[B16] Changing Nature (2013). The Economist. Available online at: http://www.economist.com/blogs/babbage/2013/02/scientific-publishing [Accessed October 22, 2015].

[B17] Clement T. P. (2014). Authorship matrix: a rational approach to quantify individual contributions and responsibilities in multi-author scientific articles. Sci. Eng. Ethics 20, 345–361. 10.1007/s11948-013-9454-3 23813053 PMC4033822

[B18] Craig I. Plume A. McVeigh M. Pringle J. Amin M. (2007). Do open access articles have greater citation impact? A critical review of the literature. J. Informetr. 1, 239–248. 10.1016/j.joi.2007.04.001

[B19] Davis M. Davis K. J. Dunagan M. M. (2012). Scientific Papers and Presentations, 3rd Edn. Amsterdam: Elsevier/AP.

[B20] Dee P. (2006). Building a Successful Career in Scientific Research: A Guide for Ph. D. Students and Post-Docs. Cambridge, UK; New York, NY: Cambridge University Press.

[B21] Desjardins-Proulx P. White E. P. Adamson J. J. Ram K. Poisot T. Gravel D. (2013). The case for open preprints in biology. PLoS Biol. 11:e1001563. 10.1371/journal.pbio.1001563 23690752 PMC3653830

[B22] Doerr A. (2013). How to write a cover letter, in Methagora Blog Nature Methods. Available online at: http://blogs.nature.com/methagora/2013/09/how-to-write-a-cover-letter.html [Accessed March 31, 2016].

[B23] Figg W. D. Dunn L. Liewehr D. J. Steinberg S. M. Thurman P. W. Barrett J. C.. (2006). Scientific collaboration results in higher citation rates of published articles. Pharmacotherapy 26, 759–767. 10.1592/phco.26.6.759 16716129

[B24] Fraser J. (2008). How to Publish in Biomedicine: 500 Tips for Success, 2nd Edn. Oxford; New York, NY: Radcliffe Pub.

[B25] Gabrys B. J. Langdale J. A. (2012). How to Succeed as a Scientist: From Postdoc to Professor. Cambridge, UK; New York, NY: Cambridge University Press.

[B26] Gould J. (2014). How to get published in high-impact journals: big research and better writing, in Naturejobs Blog. Available online at: http://blogs.nature.com/naturejobs/2014/11/03/how-to-get-published-in-high-impact-journals-big-research-and-better-writing [Accessed October 22, 2015].

[B27] Hafner J. H. (ed.). (2010). The art of the cover letter. ACS Nano 4, 2487–2487. 10.1021/nn100907e20496951

[B28] Haug C. (2013). The downside of open-access publishing. N. Engl. J. Med. 368, 791–793. 10.1056/NEJMp1214750 23445091

[B29] Janis I. L. (1972). Victims of Groupthink; a Psychological Study of Foreign-Policy Decisions and Fiascoes. Boston, MA; Houghton, MI: Mifflin.

[B30] Johnson C. Green B. (2009). Submitting manuscripts to biomedical journals: common errors and helpful solutions. J. Manipulative Physiol. Ther. 32, 1–12. 10.1016/j.jmpt.2008.12.002 19121460

[B31] Kelner K. (2007). Tips for publishing in scientific journals. Science. 10.1126/science.caredit.a0700046 Available online at: http://www.sciencemag.org/careers/2007/04/tips-publishing-scientific-journals [Accessed April 27, 2016].

[B32] Kousha K. Abdoli M. (2010). The citation impact of open access agricultural research: a comparison between OA and non-OA publications. Online Inf. Rev. 34, 772–785. 10.1108/14684521011084618

[B33] Lee S. (2005). The impact of research collaboration on scientific productivity. Soc. Stud. Sci. 35, 673–702. 10.1177/0306312705052359

[B34] Pontika N. (2015). Open Access: what's in it for me as an early career researcher? JCOM 14:C04. Available online at: http://jcom.sissa.it/archive/14/04/JCOM_1404_2015_C01/JCOM_1404_2015_C04 [Accessed March 8, 2016].

[B35] Powell K. (2010). Publications: publish like a pro. Nature 467, 873–875. 10.1038/nj7317-873a 21105223

[B36] Pronk J. T. Lee S. Y. Lievense J. Pierce J. Palsson B. Uhlen M. (2015). How to set up collaborations between academia and industrial biotech companies. Nat. Biotechnol. 33, 237–240. 10.1038/nbt.3171 25748909

[B37] Montreal Statement on Research Integrity in Cross-Boundary Research Collaborations (2013). (Montreal). Available online at: researchintegrity.org/Statements/Montreal%20Statement%20English.pdf

[B38] npg (2015). Tips for Promoting Your Research Paper. Available online at: http://www.nature.com/content/authortips/index.html?WT.mc_id=FBK_NPG_AUTHORTIPS_1509_AWARENESSPOST [Accessed October 22, 2015].

[B39] Rocha da Silva P. (2015). Selecting for impact: new data debunks old beliefs, in Frontiers Blog. Available omline at: http://blog.frontiersin.org/2015/12/21/4782/ [Accessed April 6, 2016].

[B40] Rocha da Silva P. (2016). New data debunks old beliefs: Part 2. Frontiers Blog. Available online at: http://blog.frontiersin.org/2016/03/04/initial-findings-confirmed-no-significant-link-between-rejection-rate-and-journal-impact/ [Accessed April 6, 2016].

[B41] Rosei F. Johnston T. W. (2006). Survival Skills for Scientists. London; Hackensack, NJ: Imperial College Press. Distributed by World Scientific.

[B42] Simms A. Nichols T. (2014). Social loafing: a review of the literature. J. Manag. Policy Pract. 15, 58–67.

[B43] Smalheiser N. R. Perkins G. A. Jones S. (2005). Guidelines for negotiating scientific collaboration. PLoS Biol. 3:e217. 10.1371/journal.pbio.0030217 15941361 PMC1149495

[B44] Stokols D. Misra S. Moser R. P. Hall K. L. Taylor B. K. (2008). The ecology of team science. Am. J. Prev. Med. 35, S96–S115. 10.1016/j.amepre.2008.05.003 18619410

[B45] Tscharntke T. Hochberg M. E. Rand T. A. Resh V. H. Krauss J. (2007). Author sequence and credit for contributions in multiauthored publications. PLoS Biol. 5:e18. 10.1371/journal.pbio.0050018 17227141 PMC1769438

[B46] Verma I. M. (2015). Impact, not impact factor. Proc. Natl. Acad. Sci. U.S.A. 112, 7875–7876. 10.1073/pnas.1509912112 26080449 PMC4491750

[B47] Wager E. Kleinert S. (2011). Responsible research publication: international standards for authors. A position statement developed at the 2nd World Conference on Research Integrity, Singapore, July 22–24, 2010 in Promoting Research Integrity in a Global Environment, eds Mayer T. Steneck N. (Singapore: Imperial College Press/World Scientific Publishing), 309–316.

[B48] Walker R. Rocha da Silva P. (2015). Emerging trends in peer review—a survey. Front. Neurosci. 9:169. 10.3389/fnins.2015.00169 26074753 PMC4444765

[B49] Walker R. L. Sykes L. Hemmelgarn B. R. Quan H. (2010). Authors' opinions on publication in relation to annual performance assessment. BMC Med. Educ. 10:21. 10.1186/1472-6920-10-21 20214826 PMC2842280

[B50] Wikipedia (2015). List of Collaborative Software. Wikipedia Free Encycl. Available online at: https://en.wikipedia.org/w/index.php?title=List_of_collaborative_software&oldid=686211340 [Accessed October 22, 2015].

